# Proteasome Inhibition Suppresses Dengue Virus Egress in Antibody Dependent Infection

**DOI:** 10.1371/journal.pntd.0004058

**Published:** 2015-11-13

**Authors:** Milly M. Choy, Summer L. Zhang, Vivian V. Costa, Hwee Cheng Tan, Sophie Horrevorts, Eng Eong Ooi

**Affiliations:** 1 Program in Emerging Infectious Diseases, Duke-National University of Singapore Graduate Medical School, Singapore, Singapore; 2 Interdisciplinary Research Group in Infectious Diseases, Singapore-MIT Alliance for Research and Technology, Singapore, Singapore; 3 Department of Molecular Cell Biology and Immunology, VU University Medical Center, Amsterdam, The Netherlands; Johns Hopkins Bloomberg School of Public Health, UNITED STATES

## Abstract

The mosquito-borne dengue virus (DENV) is a cause of significant global health burden, with an estimated 390 million infections occurring annually. However, no licensed vaccine or specific antiviral treatment for dengue is available. DENV interacts with host cell factors to complete its life cycle although this virus-host interplay remains to be fully elucidated. Many studies have identified the ubiquitin proteasome pathway (UPP) to be important for successful DENV production, but how the UPP contributes to DENV life cycle as host factors remains ill defined. We show here that proteasome inhibition decouples infectious virus production from viral RNA replication in antibody-dependent infection of THP-1 cells. Molecular and imaging analyses in β-lactone treated THP-1 cells suggest that proteasome function does not prevent virus assembly but rather DENV egress. Intriguingly, the licensed proteasome inhibitor, bortezomib, is able to inhibit DENV titers at low nanomolar drug concentrations for different strains of all four serotypes of DENV in primary monocytes. Furthermore, bortezomib treatment of DENV-infected mice inhibited the spread of DENV in the spleen as well as the overall pathological changes. Our findings suggest that preventing DENV egress through proteasome inhibition could be a suitable therapeutic strategy against dengue.

## Author Summary

The lack of either licensed vaccine or antiviral drug has resulted in approximately 400 million dengue infections annually. A possible rapid approach to a specific therapeutic for dengue is to use a licensed inhibitor of a host factor critically required by dengue virus (DENV) to complete its life cycle. One such set of factors is in the ubiquitin proteasome pathway (UPP). Despite the availability of licensed proteasome inhibitors, these studies have not led to any clinical translation, because the mechanism of action of this pathway on the virus life cycle is uncertain. We demonstrate that the UPP is critical for DENV egress after replication in human target cells. Intriguingly, treatment with the licensed proteasome inhibitor, bortezomib, inhibited the overall pathological changes in wild-type mice. Altogether, our study provides new insights into the role a functional UPP plays in DENV infection and suggests a potential therapeutic strategy against dengue by repurposing a licensed drug.

## Introduction

Dengue has emerged to be the most important mosquito-borne viral disease globally. An estimated 390 million infections occur annually while another 3 billion people that live in or travel to the tropics are at constant risk of infection with any of the four dengue virus (DENV) serotypes [1]. While the effort to develop a licensed vaccine appears to have taken significant strides recently [2,3], whether vaccination can produce long-lasting protection against all virus serotypes remains to be determined. An important consideration is whether vaccination can avoid antibody-enhanced infection that is epidemiologically associated with increased risk of severe dengue [4,5]. Consequently, effective antiviral therapies against dengue would not only address disease burden imposed by dengue, it would also be useful in vaccinated populations should vaccine failure occur. Antiviral therapies must also be effective against both primary and secondary infections; the latter may be enhanced by the presence of heterologous antibodies and is associated with increased risk of severe disease.

A rapid approach to therapeutic development is to repurpose existing licensed drug [6–8]. Indeed, DENV relies on host factors to supplement their relatively simple genome [9–12]. Hence, drugs that inhibit critical host factors could effectively stall the completion of the virus life cycle. Functional genomic screens as well as basic and clinical studies has identified several important host factors in the ubiquitin-proteasome pathway (UPP) [13–15]. This pathway is an attractive target for several reasons. Firstly, drugs that inhibit function of the proteasome, a major player of the UPP, have been licensed for therapeutic use. Secondly, genes in this pathway have been found to be differentially expressed during DENV infection [13,14,16] and serve as flaviviral replication promoting factors [10,11]. Thirdly, pharmacological inhibition of the UPP, such as proteasome inhibition [13] or interference with the ubiquitin E1 activity [14] has been shown to reduce DENV production significantly, *in vitro*. However, how the UPP serves to promote DENV replication remains ill defined. One possibility is that proteasome inhibition blocks DENV entry via endocytosis [11,17], which is dependent on ubiquitylation [18]. However, this process may be cell-type dependent [19]. Furthermore, when opsonized with non- or sub-neutralizing levels of antibody, DENV is also able to bypass endocytosis and enter monocytes, macrophages, and dendritic cells, which are the primary targets of DENV via proteasome independent Fc receptor-mediated phagocytosis [20]. Thus, if proteasome inhibition only inhibits viral entry, it would not be a suitable therapeutic strategy for antibody-enhanced DENV infection.

In this report, we investigated if proteasome inhibition can inhibit other parts of the viral life cycle besides viral entry. Using antibody-dependent infection of monocytic cells as a tool to bypass viral entry via endocytosis, our data suggests that a functional UPP is required for DENV egress. Finally, we demonstrate *in vitro* with primary monocytes and *in vivo* with a mouse infection model that virus replication is exquisitely sensitive to proteasome inhibition. Such a therapeutic approach may apply to other viruses that rely on a functional proteasome to complete their life cycle.

## Results

### Proteasome inhibition decouples infectious DENV2 production from viral RNA replication in THP-1 cells

To elucidate the role of the proteasome on DENV2 replication, we took advantage of a subclone of THP-1 human monocytic cells for our investigations [21]. Importantly, inhibition of the proteasome could potentially inhibit virus entry via endocytosis [11]. This potential confounder can be bypassed by opsonizing DENV with enhancing levels of antibody where virus entry via proteasome independent Fc receptor-mediated phagocytosis can occur [20].

**Fig 1:**
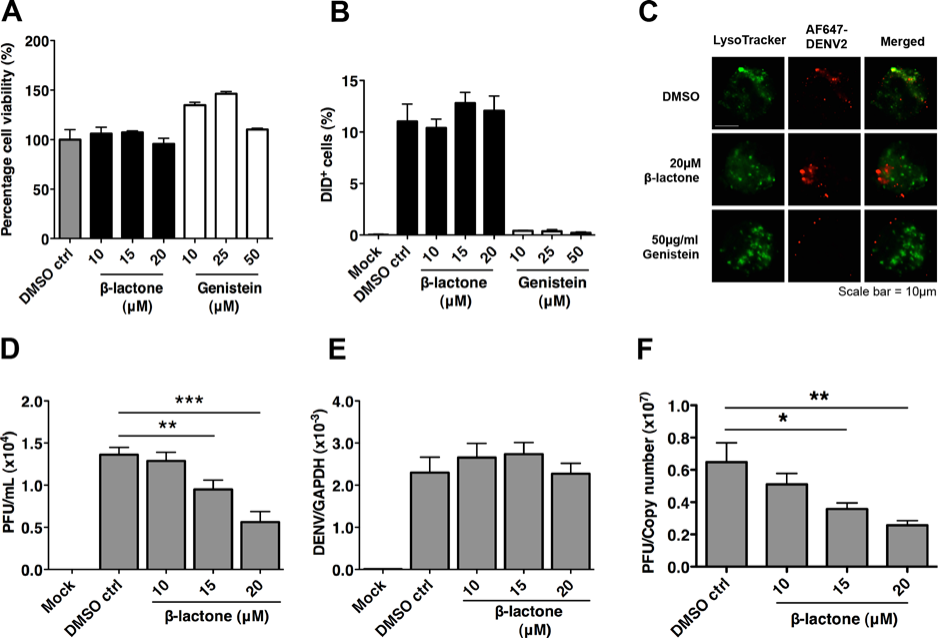
Proteasome inhibition decouples infectious DENV2 production from viral RNA replication in THP-1 cells. (A) No cytotoxicity was observed in THP-1 cells in the presence of β-lactone and genistein for 48 h using a cell viability assay. Mean ± SD. N = 4. (B) Using flow cytometry, β-lactone does not block uptake of DENV2 immune complexes. Genistein, an inhibitor of Fc receptor-mediated entry blocked uptake. (C) Alexa Fluor 647 labeled DENV2 opsonized with h3H5 were internalized in cells pre-treated with DMSO or β-lactone, while genistein prevented the uptake of virus at 2 hpi. (D) Proteasome inhibition showed a dose-dependent decrease in plaque titers 48 hpi. Mean ± SD. N = 4. Student’s t test, **p<0.01, ***p<0.001. (E) DENV2 RNA copy number per GAPDH showed no significant difference between β-lactone treated and DMSO control 48 hpi. (F) Ratio of infectious DENV2 to genomic copies showed a dose-dependent decrease after β-lactone treatment. Mean ± SD. N = 4. Student’s t test, *p < 0.05, **p<0.01.

To demonstrate that proteasome inhibition with clasto lactacystin β-lactone (β-lactone), a widely used proteasome inhibitor, indeed did not alter virus entry at non-toxic levels (Fig 1A), we measured DiD (1,1'-dioctadecyl-3,3,3',3'-tetramethylindodicarbocyanine, 4-chlorobenzenesulfonate salt)-labeled DENV in β-lactone treated cells. DENV2 opsonized with enhancing levels of humanized 3H5 monoclonal antibody (h3H5) was added to a THP-1 subclone with increased susceptibility to antibody-dependent infection [21]. Cells were pretreated with β-lactone or DMSO as a vehicle control. Results showed that the proportion of DiD-positive cells were similar to that of DMSO control (Fig 1B). As a positive control, we treated cells with genistein, a specific tyrosine kinase inhibitor that blocks Fc receptor-mediated phagocytosis [22,23]. Genistein treatment significantly reduced uptake of h3H5-opsonized DENV in a concentration-dependent manner (Fig 1B) [24]. Similar observations were made with confocal microscopy where Alexa Fluor 647 labeled DENV [25] opsonized with h3H5 were internalized in cells pre-treated with DMSO or β-lactone, while genistein prevented the uptake of virus at 2 hours post infection (hpi) (Fig 1C). Altogether, these data indicate that any antiviral effect observed during β-lactone treatment is independent of DENV entry.

While no difference was observed in the proportion of cells infected with fluorophore-labeled DENV2, the outcome of infection was significantly different. Treatment with β-lactone resulted in a significant dose-dependent reduction in infectious DENV2 titers, measured by plaque forming units (PFU) (Fig 1D) despite no observable decrease in viral genomic RNA levels 48 hpi (Fig 1E). Correspondingly, the ratio of PFU to intracellular DENV2 genome decreased after β-lactone treatment (Fig 1F). These findings suggest that proteasome inhibition does not inhibit antibody-dependent infection but instead decouples infectious virus production from DENV2 genome replication.

### DENV2 egress is dependent on proteasome function

**Fig 2:**
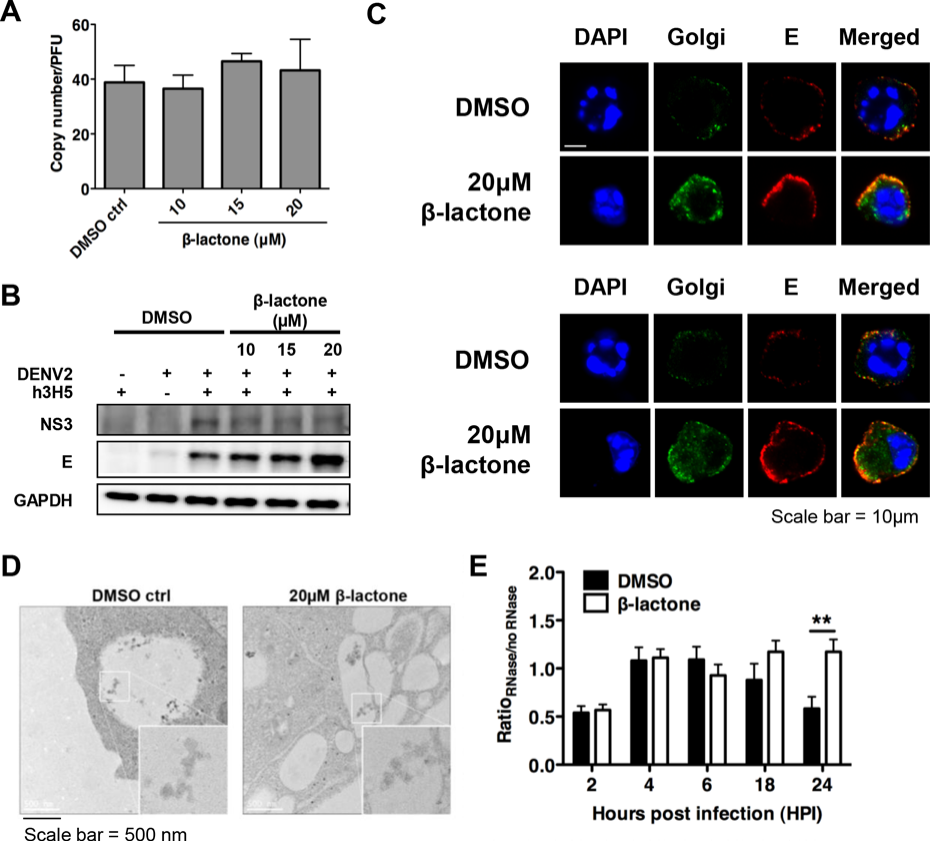
DENV2 egress is dependent on proteasome function. (A) Ratio of viral genomic RNA from cell culture supernatant to infectious DENV2 showed no significant differences after β-lactone treatment at different drug concentrations, compared to DMSO control, suggesting that the treatment with β-lactone did not result in reduced DENV2 maturation. Mean ± SD. N = 4. (B) A dose-dependent accumulation of E protein in cells is observed with no difference in the levels of NS3 at 24 hpi. (C) Confocal analysis showed accumulation of structural proteins (prM and E, in red) in β-lactone treated compared to DMSO treated cells at 24 hpi with strong co-localization with Golgi (in green), suggesting that egress of viral particles were impaired in presence of UPP inhibition (Scale bar = 10 μm). (D) Accumulation of viral particles in intra-cytoplasmic vacuoles (50 nm) in β-lactone compared to DMSO treated cells was observed using electron microscopy, indicating that DENV2 RNA could replicate and be packaged with structural proteins (Scale bar = 500 nm). (E) Ratio of RNase to non-RNase treated cells after β-lactone treatment is significantly higher compared to the DMSO control 24 hpi. Mean ± SD. N = 4. Student’s t test, **p<0.01.

The observed decoupling of infectious DENV production from RNA replication in β-lactone treated cells has three possible explanations. Firstly, newly formed DENV were released from the cells, but as immature and non-infectious particles. Alternatively, normal proteasome function may be critical for either virus assembly or egress from infected cells. To test the first possibility, we measured viral RNA in the cell culture supernatant by qRT-PCR and compared it with the amount of infectious virions [26]. Ratio of viral genomic RNA to PFU showed no statistically significant difference regardless of the drug concentration used (Fig 2A), indicating that treatment with β-lactone did not result in the release of proportionately more immature DENV compared to DMSO treated cells.

To test whether UPP is essential for virion assembly or egress, we measured the amount of viral proteins or particles in infected cells. Results showed a dose-dependent increase in the DENV E protein with no change in NS3 protein on a western blot in β-lactone treated cells 24 hpi (Fig 2B). Confocal microscopy also showed similar accumulation of structural proteins, E and prM in β-lactone compared to DMSO treated cells at 24 hpi with strong co-localization with Golgi markers (Fig 2C). Electron microscopy revealed 50 nm viral particles in intra-cytoplasmic vacuoles in both β-lactone or DMSO treated cells, suggesting that DENV RNA could replicate, and be translated and packaged with structural proteins to form virions (Fig 2D).

To test quantitatively the possibility that packaged virions accumulate in the cell upon proteasome inhibition, we harvested β-lactone or DMSO treated cells at 2, 4, 6, 18 and 24 hpi. The cells were lysed and treated with RNase to remove any RNA, except those packaged into virions (S1A and S1B Fig) which were then measured using qRT-PCR. The ratio of viral genome copy equivalents measured with or without RNase treatment provided an indication of the proportion of viral genome that was packaged into virions. In DMSO control, the proportion of packaged viral RNA peaked at 6 hpi and declined thereafter, consistent with the notion that DENV egresses from cells upon completion of replication (Fig 2E). With β-lactone treatment, however, the proportion of packaged viral RNA remained elevated up till 24 hpi (Fig 2E). Collectively, these results suggest that the UPP is critical for DENV egress from the host cell for subsequent rounds of infection.

### Proteasome inhibition increases eIF2α phosphorylation and represses translation of EXOC7, TC10 and EXOC1

**Fig 3:**
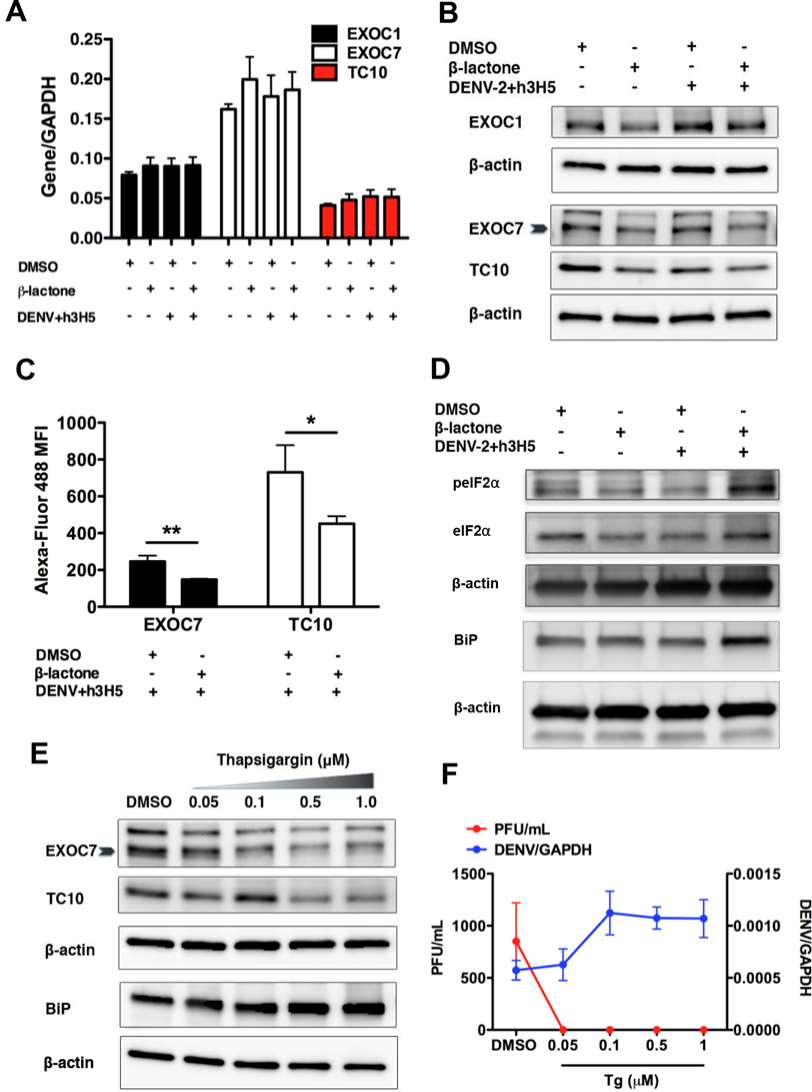
Proteasome inhibition increases eIF2α phosphorylation and represses translation of EXOC7, TC10 and EXOC1. Effects of proteasome inhibition on the expression levels of EXOC7, its effector, TC10, and its interacting partner, EXOC1 were assessed. (A) Transcript levels of EXOC7, TC10 and EXOC1 remained constant. Mean ± SD. N = 4. (B) Protein levels of EXOC7, TC10 and EXOC1 decreased in both uninfected and DENV2-infected β-lactone treated cells. (C) Using flow cytometry, the mean fluorescence intensity of EXOC7 and TC10 decreased significantly i7n β-lactone treated infected cells compared to DMSO treated infected cells, indicating a decrease in the protein levels of EXOC7 and TC10 after β-lactone treatment. Mean ± SD. N = 4. Student’s t test, *p<0.05, **p<0.01. (D) Effect of proteasome inhibition on eIF2α phosphorylation was assessed in DMSO or β-lactone treated infected THP-1 cells. Increased phosphorylation of eIF2α was observed in β-lactone treated infected cells 4 hpi. Up-regulation of the resident ER chaperone protein BiP in DENV2-infected β-lactone treated cells was also observed. (E) ER stress inducer, thapsigargin, reduced protein levels of EXOC7 and TC10 in a dose-dependent manner. BiP, an ER stress signature, increases with increasing thapsigargin concentration. (F) Similar to DENV2-infected β-lactone treated cells, DENV2-infected thapsigargin treated THP-1 cells showed the same decoupling effect of infectious particles to RNA copy number. Viral RNA genome was detected in thapsigargin treated cells but no infectious DENV2 was detected in cell culture supernatant using plaque assay. Mean ± SD. N = 4.

DENV completes its life cycle by egressing from host cells through exocytosis modulated by the exocyst complex. EXOC7, a component of the exocyst complex is involved in the docking of exocytic vesicles with fusion sites on the plasma membrane via interaction with TC10, a Rho GTPase [27]. More recently, EXOC7 has been shown in particular to be critical for DENV egress [28]. We thus investigated the effects of proteasome inhibition on the expression levels of EXOC7, its effector, TC10, and its interacting partner, EXOC1. Our results indicate that transcript levels of EXOC7, TC10 and EXOC1 remained constant after proteasome inhibition (Fig 3A). Interestingly, the levels of the corresponding proteins decreased moderately in β-lactone treated cells, under both DENV2-infected and uninfected conditions (Fig 3B). The differences in protein levels of EXOC7 and TC10 between DMSO and β-lactone treated DENV-infected cells were statistically significant when measured by flow cytometry (Fig 3C).

A mechanism that is known to repress translation is the ER stress-induced eIF2α-mediated translational repression of cellular mRNA. eIF2α is an effector of the PKR-like ER kinase (PERK) pathway in the UPR (unfolded protein response) and phosphorylation of this protein prevents GDP-GTP exchange on eIF2α by the guanine nucleotide exchange factor eIF2B, thereby inhibiting recycling of the ternary complex that contains the initiator methionine Met-tRNAi [29–31]. Consequently, global translation initiation, including that of EXOC7 and TC10, is decreased. We thus tested if proteasome inhibition could activate the PERK pathway. eIF2α phosphorylation was assessed in β-lactone treated THP-1 cells with DENV2 infection. As expected, increased phosphorylation of eIF2α was observed in DENV2-infected β-lactone treated cells at 4 hpi (Fig 3D). Up-regulation of the resident ER chaperone protein BiP, an ER stress protein, in DENV2-infected β-lactone treated cells was also observed at 4 hpi (Fig 3D). These findings suggest the combination of DENV infection and proteasome inhibition may increase ER stress.

If translational repression due to ER stress explains our observations, treatment of THP-1 cells with an ER stress agonist such as thapsigargin should lead to a similar reduction in EXOC7 and TC10 levels as well as DENV egress. Indeed, EXOC7 and TC10 levels in THP-1 cells showed a dose-dependent reduction with thapsigargin treatment whereas BiP level increased with increasing thapsigargin concentration, with no observable cytotoxicity to the cells (Figs 3E and S2A). Likewise, although the virus genome was detected in thapsigargin treated DENV2-infected cells, no infectious DENV2 was detected using plaque assay (Fig 3F). Taken collectively, our results suggest that UPP inhibition increases ER stress, which may trigger the UPR. Downstream activation of PERK can then attenuate the translation of exocyst complex components, which may be required for dengue egress via exocytosis.

### Bortezomib inhibits infectious DENV production in primary monocytes

**Fig 4:**
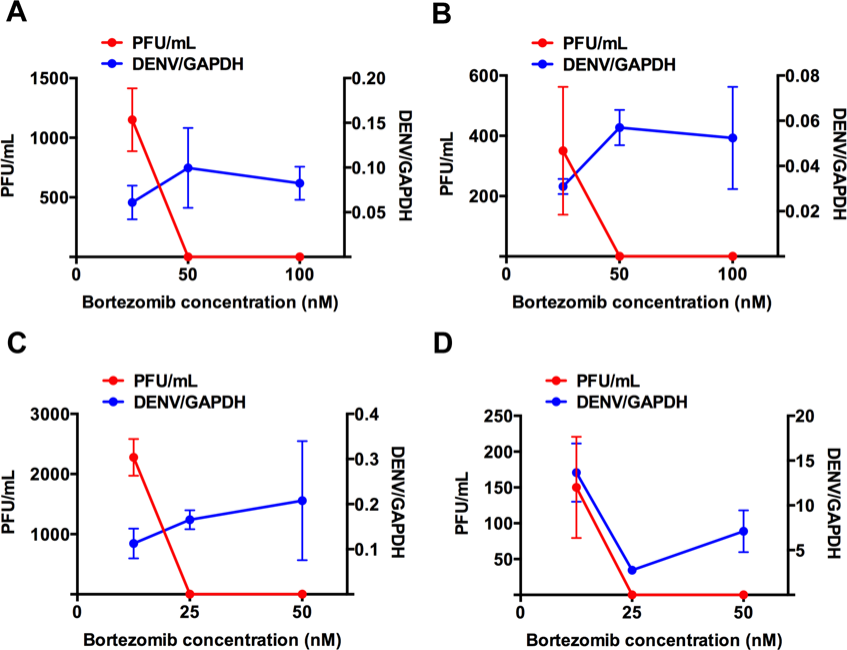
Bortezomib decouples infectious DENV production from viral RNA replication in primary monocytes. Viral RNA genome was detected using qRT-PCR, but no infectious DENV2 was detected using plaque assay in the supernatant of cells treated with higher concentrations of bortezomib for (A) DENV1, (B) DENV2, (C) DENV3 and (D) DENV4. Mean ± SD. N = 4.

**Fig 5:**
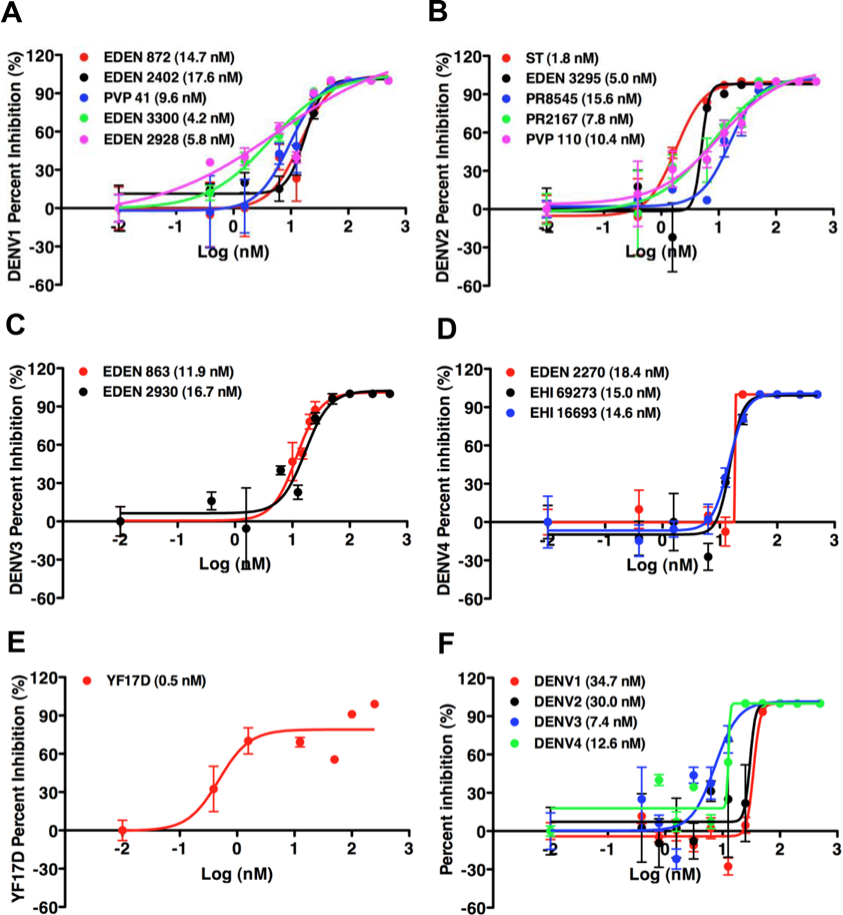
Bortezomib inhibits infectious DENV production in primary monocytes. Infectious DENV in cell culture supernatant was measured using plaque assay, and the percent inhibition of infectious DENV production after bortezomib treatment compared to DMSO treatment was calculated. At doses of bortezomib that is minimally toxic to primary monocytes, a significant dose-dependent decrease in virus titers was observed for different strains of all 4 dengue serotypes, (A) DENV1, (B) DENV2, (C) DENV3 and (D) DENV4, after drug treatment in primary monocytes. The identity of the clinical isolate is indicated next to a distinct colored dot. The bortezomib concentration that inhibited 50% of virus replication (in parentheses) was less than 20 nM for DENV1, 2, 3 and 4, respectively. (E) Bortezomib also inhibited 50% of virus production of the attenuated strain of yellow fever virus, YF17D, at a concentration of 0.5 nM. (F) Epoxomicin, another proteasome inhibitor, was able to reduce DENV titers by plaque assay in a dose-dependent manner for all 4 dengue serotypes. Mean ± SD. N = 4.

To ensure that our findings are not limited to THP-1 cell line or the use of β-lactone, we also explored if bortezomib, a FDA-approved reversible proteasome inhibitor used to treat multiple myeloma and mantle cell lymphoma, could inhibit DENV egress in primary monocytes at doses that cause minimal cytotoxicity. The 50% cytotoxic concentration (CC50) of bortezomib in primary monocytes is above 1 μM (S2B Fig). DENV1-4 opsonized with enhancing levels of humanized 4G2 (h4G2) monoclonal antibody was used to infect primary monocytes [21]. Indeed, although the virus genome was detected in bortezomib treated DENV-infected cells for all four serotypes of DENV, no infectious DENV was detected using plaque assay at higher concentrations of bortezomib (Fig 4A–4D). Furthermore, pre-treatment of primary monocytes with bortezomib also inhibited replication of different low-passage clinical isolates of all 4 serotypes of DENV in a dose-dependent manner (Fig 5A–5D). The maximal effective concentration of bortezomib that inhibited 50% of virus replication (EC_50_) is less than 20 nM for each of these isolates. Bortezomib was also able to inhibit 50% of virus production of the attenuated strain of yellow fever virus, YF17D, at a concentration of 0.5 nM, suggesting the dependence of functional UPP is not limited to DENV but may also apply to other flaviviruses (Fig 5E). Similar observations were also made when another proteasome inhibitor, epoxomicin, from which the licensed carfilzomib was derived, was used at concentrations well tolerated by primary monocytes (Figs 5F and S2C).

### Bortezomib reduced viral load and signs of dengue pathology in C57BL/6 mice

The ability of proteasome inhibition to prevent completion of the DENV life cycle at low nanomolar concentrations suggests that this class of drug could have therapeutic potential for dengue patients. To test this possibility, we adopted a recently reported immunocompetent animal model [32] to test the *in vivo* efficacy of bortezomib as an anti-dengue drug. C57BL/6 mice were chosen in this study as they display several signs upon DENV infection, such as thrombocytopenia and elevated hematocrit levels, which are consistent with those observed in human dengue cases. Importantly, these animals are also immunocompetent, which could be necessary to deal with DENV entrapped in infected cells due to inhibition of egress.

**Fig 6:**
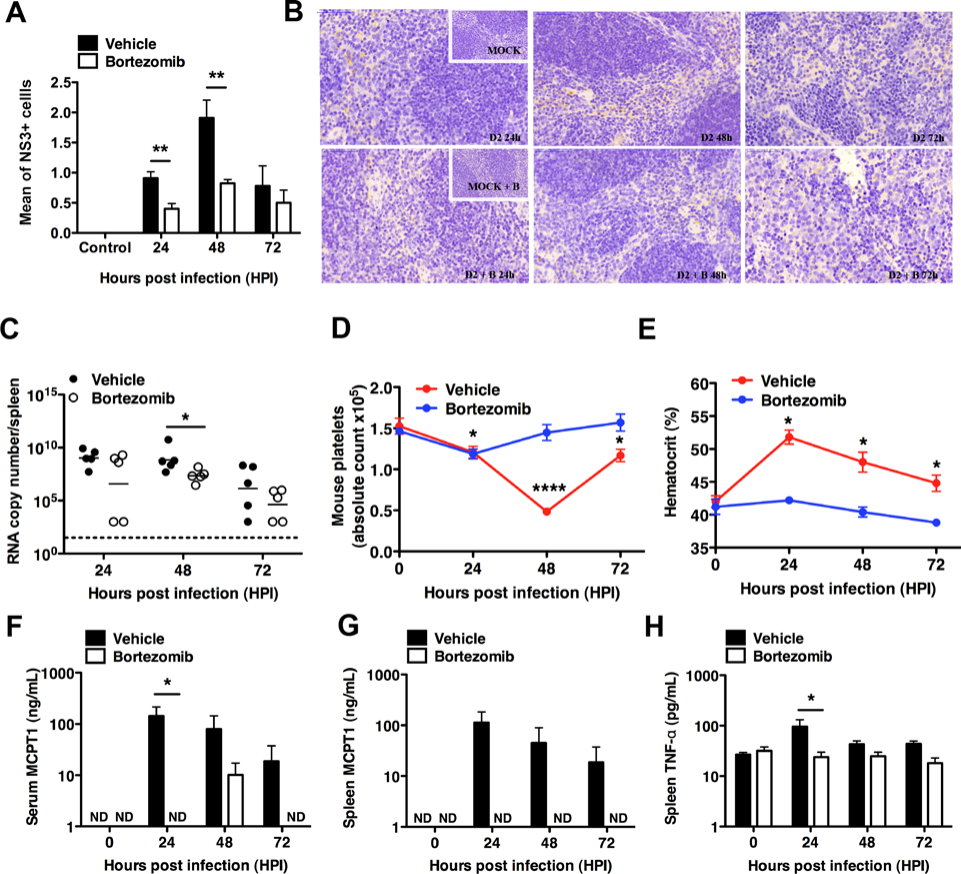
Bortezomib reduced viral load and signs of dengue pathology in C57BL/6 mice. WT mice infected intraperitonealy with DENV2 were treated with bortezomib 6 hpi and analyzed at 24, 48 and 72 hpi for further analysis. (A) For quantification of NS3+ cells, the mean cell count in 20 alternate microscopic high-power fields (x400) was measured. Quantification was done only in the red pulp of spleen. Bortezomib treated mice showed significantly reduced number of DENV infected cells in the red pulp of the spleen at 24 and 48 hpi compared to vehicle control. Mean ± SEM. N = 4–5. Student’s t test, **p<0.01. (B) Representative serial sections from spleen of each group of mice stained with anti-DENV NS3 antibody at 24, 48 and 72 hpi. Multiple sections of each tissue were examined for staining (60x magnification). The inset is a representation of the spleen from mock infected mice without (top panel) or with (bottom panel) bortezomib treatment. The top panel shows DENV-infected spleen without bortezomib treatment 24, 48 and 72 hpi. The bottom panel shows DENV-infected spleen with bortezomib treatment 24, 48 and 72 hpi. Positive staining for NS3 is brown while hematoxylin counterstaining is blue. Bortezomib treated mice also showed significantly reduced number of DENV infected cells in the red pulp of the spleen at 24 and 48 hpi compared to vehicle control. (C) RNA copy number in the spleen was reduced in bortezomib treated mice compared to vehicle control at 48 hpi. Mean ± SEM. N = 4–5. Student’s t test, *p<0.05. (D-E) The vehicle control experienced a drop in platelet count over the first 48 hours of infection before recovering by day 3, and experienced a significant rise in hematocrit values that peaked 24 hpi. On the other hand, no significant changes were observed for the platelet count 24–72 hpi, and hematocrit levels 0–72 hpi in mice after bortezomib treatment, suggesting the efficiency of bortezomib in alleviating disease symptoms. Mean ± SEM. N = 4–5. Student’s t test, *p<0.05, ****p<0.0001. (F-G) DENV2-infected vehicle control displayed an increased systemic level of MCPT1, indicative of mast cell activation, in mouse serum and spleen when compared to bortezomib treated mice at all time-points. (H) Levels of TNF-α were decreased in bortezomib treated mice 24 hpi when compared to the vehicle control. Mean ± SEM. N = 4–5. Student’s t test, *p<0.05.

We treated C57BL/6 mice infected with DENV2 with a single dose of bortezomib at 6 hpi. The dose was based on that licensed for the treatment of multiple myeloma. The spleen was chosen for analysis since previous work has shown that this animal model produces no detectable viremia but the viral load in the spleen correlated with the degree of plasma leakage [32]. Using immunohistochemistry, we observed that mice treated with bortezomib showed significantly reduced number of DENV infected cells in the red pulp of the spleen at 24 and 48 hpi compared to vehicle control (Fig 6A and 6B). No difference in NS3-positive cells was observed at 72 hpi, which is consistent with previously reported data that this strain of mice clears DENV infection rapidly without intervention [32]. Consistently, a significant difference in viral RNA genome copies was also observed at 48 hpi in bortezomib treated cells (Fig 6C). Both observations suggest that proteasome inhibition could inhibit virus egress and hence spread in mammals. Along with reducing viral burden, bortezomib treatment also reduced the degree of thrombocytopenia (Fig 6D) and plasma leakage (Fig 6E) compared to control animals.

Besides reducing the degree of change in platelet count and hematocrit, bortezomib treatment also reduced the previously observed inflammatory response in infected mice [32]. MCPT1 (Mouse Mast Cell Protease-1), an indicator of mast cell activation, was not detected in both serum and spleen in bortezomib treated mice compared to controls (Fig 6F and 6G). Levels of TNF-α were also decreased in bortezomib treated mice compared to the vehicle control (Fig 6H). These findings collectively indicate that bortezomib treatment is able to reduce DENV2 replication and the subsequent pro-inflammatory response, *in vivo*.

## Discussion

The UPP is a major extra-lysosomal pathway for regulated protein degradation, clearing misfolded or obsolete proteins and maintaining protein homeostasis. The proteasome is the main driver of the UPP as it recognizes and degrades polyubiquitylated proteins, modified via covalent attachment of ubiquitin through the sequential activities of E1-activating, E2-conjugating, and E3 ligase enzymes [33]. In this study, we show that proteasome inhibition does not prevent DENV replication but rather virus egress, in antibody-dependent infection of monocytic cells. Egress from the infected cell is perhaps one of the most ill-defined parts of the DENV life cycle. Previous work has demonstrated that this process occurs through exocytosis and is hence dependent on the expression levels of component of the exocyst complex [28].

The mechanism in which the UPP affects DENV egress remains to be determined. While there could be other mechanisms involved, our study raises the possibility that the expression of the exocyst component, EXOC7, and its effector, TC10, is regulated at the translational level, at least in part, by ER stress response. One explanation is that to ensure successful completion of its life cycle, DENV may rely on the proteasome to alleviate ER stress. Inhibition of the proteasome results in the accumulation of misfolded or obsolete proteins [34], which induces the ER stress response [35,36], triggering the PERK pathway in the UPR that represses translation of the exocyst components needed for exocytosis. In our study, although the levels of EXOC7 and TC10 decreased moderately in β-lactone treated cells, inducing ER stress with an agonist without inhibiting proteasome function recapitulated the observed down-regulation of EXOC7 and TC10 protein levels, along with the decoupling of infectious virus production from viral RNA replication. Supporting our data, salubrinal, a drug that inhibits eIF2α dephosphorylation, thereby increasing phosphorylated eIF2α levels was previously shown to reduce the production of infectious viruses [37].

While we have demonstrated the inhibition of virus egress as the antiviral mechanism effected by proteasome inhibition, it is interesting that this drug may have other modes of antiviral action. The UPP has also been shown to be critical for the life cycle of Nipah virus. Inhibition of the proteasome led to impaired nuclear export of the viral matrix protein to the cytoplasm [38]. The authors showed that a conserved lysine residue on this protein required mono-ubiquitylation for nuclear export. Concomitantly, depletion of free ubiquitin through proteasome inhibition inhibited the Nipah virus life cycle with exquisite sensitivity [38]. Likewise, studies on retroviruses have also demonstrated that disruption of the proteasome function depletes the free ubiquitin pool [39], which is necessary for the ubiquitylation of late domain on Gag protein for proper viral budding [40,41]. While such mechanisms could also contribute to our observed inhibition of DENV replication, there is as yet no evidence that the function of any of the DENV proteins need to be activated by mono-ubiquitylation [14].

Although proteasome inhibition has been shown to exhibit anti-DENV activity in other cell types [13,14], the effect of inhibiting endocytosis through proteasome inhibition appears to be cell strain specific, where HeLa cells from different laboratories respond to proteasome inhibitors differently with regard to viral endocytosis [11, 19]. This may apply to the effect proteasome inhibition has on virus egress as well. However, we have focused our investigations on monocytic cells since this cell type has been shown to be important clinically in supporting DENV replication [42, 43]. Likewise, post mortem histopathological analyses showed evidence of replicating DENV (from expression of NS3) in hepatocytes and Kupffer cells in the liver and in macrophage-like cells in the spleen and lymph nodes. No virus was detected in endothelial cells in any organs examined [43, 44].

We have focused our attention on the UPP as small molecule inhibitors of the proteasome have been licensed for use and could potentially be repurposed as a treatment for dengue. That proteasome inhibition could inhibit virus entry by both endocytosis, and egress by exocytosis would thus prevent both antibody-independent and antibody-enhanced infection. Indeed, the potency of proteasome inhibition as an anti-dengue strategy is suggested by the low nanomolar EC_50_ of bortezomib in DENV-infected primary monocytes. Similarly, bortezomib treatment in an immunocompetent mouse model was able to reduce both viral burden and pathological hallmarks of dengue. The potential of this drug to serve as an anti-dengue therapy needs to be explored in clinical safety and efficacy trials. Indeed, a known side effect of bortezomib is thrombocytopenia, although this is only observed in multiple myeloma patients after weeks of continuous treatment. As treatment for dengue would not exceed a week, the side effects observed only after prolonged therapy may not be relevant for dengue. One limitation, however, is that bortezomib is given subcutaneously or intravenously to patients. This is not ideal for any anti-dengue therapeutics, as injections are not recommended for dengue patients having the tendency to bleed. Opportunely, this problem can be circumvented by the recent introduction of ixazomib, the first oral proteasome inhibitor that is currently undergoing Phase 3 clinical studies [45].

Finally, another challenge that this and other antiviral therapies will face is how soon after the onset of illness must the drug be administered to produce the desired therapeutic effect. As the early features of dengue are mostly indistinguishable from other acute febrile illness, the remaining viremic period may be very short by the time a confirmatory dengue diagnosis is made. The effect of antiviral therapies may thus have minimal or even no efficacy at that point in the course of illness. Therefore, rapid point-of-care diagnostic tests that can reliably differentiate dengue from other causes of acute febrile illness should be prioritized for development to complement the advances in therapeutic development for dengue.

In conclusion, our study provides new insights into the UPP plays in DENV infection, and suggests a potential therapeutic strategy against dengue by repurposing a licensed drug.

## Materials and Methods

### Ethics statement

This study was carried out in strict accordance with the institutional ethical guidelines of the National University of Singapore (NUS) Government's ethical and animal experiments regulations. All experiments involving mice were performed in compliance with the guidelines of the institutional committee at NUS. The Institutional Animal Care and Use Committee (IACUC) approved the experimental protocol (IACUC, Permit Protocol Number 2013–06157). All efforts were made to minimize suffering. The guidelines followed by this Committee are based on the guidelines of Animal Welfare Act (AWA) and associated Animal Welfare Regulations (AWRs) and Public Health Service (PHS) Policy.

### Cells

THP-1 cells, BHK-21, Vero and C6/36 cell lines were purchased from the American Type Culture Collection (ATCC) and cultured according to ATCC recommendation. Peripheral blood mononuclear cells were isolated from principal investigator’s blood using Ficoll-hypaque (GE Healthcare) and plastic adhered to obtain primary monocytes. The primary monocytes were then allowed to recover overnight before use in experiments.

### Virus stock

Different strains of DENV1 (EDEN 872, EDEN 2402, EDEN 2928, EDEN 3300 and PVP41), DENV3 (EDEN 803, EDEN 863 and EDEN 2930) and DENV4 (EDEN 2270, EHI 69273 and EHI 16693) used in this study are clinical isolates from Singapore, while different strains of DENV2 (ST, EDEN 3295, PR2167, PR8545 and PVP110) used are clinical isolates from Singapore and Puerto Rico. DENV was propagated as detailed in S1 File and infectious titer was determined by plaque assay as detailed in S1 File.

### DENV2 infection in monocytes

THP-1 cells were pretreated for 1 h with DMSO or stated concentrations of β-lactone (Sigma Aldrich) or thapsigargin (Sigma Aldrich) before addition of DENV2 (moi 10) opsonized with enhancing concentrations of humanized 3H5 antibodies (0.39 μg/mL). The mixture was then incubated for 20 min on ice to synchronize entry and infection was performed for 2 h at 37°C. The cells were then washed thrice in PBS to remove any inoculum that was not phagocytosed and cultured in maintenance media for another 46 h. Cells and supernatants were harvested for qRT-PCR using 3’UTR dengue primers and GAPDH as control, and plaque assay analyses. For bortezomib and epoxomicin experiment, primary monocytes were pretreated with stated concentrations of bortezomib diluted in PBS or epoxomicin diluted in DMSO and infected with DENV1-4 (moi 10) opsonized with enhancing concentrations of humanized 4G2 antibodies (1.56 μg/mL). The supernatants were harvested at 48 h for plaque assay analyses.

### Molecular and imaging analyses of DENV2 egress

Western blot, confocal microscopy and electron transmission microscopy as detailed in S1 File was used to study DENV2 egress.

### RNase treatment of β–lactone treated cells

DENV2-infected THP-1 cells were harvested at different time-points after infection. Two freeze-thaw cycles were performed to lyse the cells. The lysates were divided into two equal aliquots and to one set of the aliquots, 1 μ1 of RNase A/T1 Cocktail Enzyme Mix (Ambion) was added to degrade any host and unpackaged viral RNA. After incubation for 30 min at 37°C, viral RNA extraction (QIAamp Viral RNA Mini Kit, Qiagen) and qRT-PCR was performed to quantify the amount of packaged DENV2 in the cells.

### Flow cytometry analysis

THP-1 cells were washed 3 times with PBS and fixed with 3% paraformaldehyde. The cells were then washed 3 times with FACS buffer consisting of PBS with 0.1% fetal bovine serum and permeabilized with 0.1% saponin in PBS. Cells were subsequently double-stained for the presence of DENV using anti-DENV complex monoclonal antibody, MAB8705, and anti-EXOC7 antibody (Abcam) or anti-TC10 antibody (Abcam) before reading on BD LSRFortessa machine and analyzed with BD FACSDiva software.

### Bortezomib treatment in DENV-infected C57BL/6 mice

C57BL/6 mice were obtained from *In Vivos* company and maintained at MD2 facility of NUS. All experiments were performed in compliance with the guidelines of the institutional committees at NUS and Singapore-Massachusetts Alliance Institute of Technology (SMART Centre). Briefly, eight to ten week-old male mice were inoculated with 1 x 10^6^ PFU of DENV2 EDEN3295 intraperitonealy (i.p) as previously described in [32]. Six hours after DENV2 infection, mice were treated with a single dose of bortezomib (1 mg/kg) via subcutaneous route (s.c) diluted in PBS solution. Vehicle mice received only PBS. Euthanasia was performed 24, 48 or 72 h after DENV2 inoculation. During all time point, the hematocrit level and platelet count in whole blood were analyzed as detailed in S1 File. MCPT-1 quantification in serum and spleen, and TNF-α levels in spleen were performed using commercially available ELISA assays (Ebioscience and R&D Systems, Minneapolis, MN, respectively) in accordance with the manufacturer’s instructions. For viral load quantification, 30 mg of spleen was collected and stored in RNAlater stabilization reagent (Qiagen) at -20°C. Spleens were homogenized using stainless steel beads (5mm) in Qiagen TissueLyser LT. The homogenate was collected, and viral RNA extraction for qRT-PCR was performed as detailed in S1 File.

### Immunohistochemistry analysis of mouse spleen

Immunohistochemistry analyses for detection and quantification of DENV infected cells in the spleen from the mice were performed. After euthanasia, spleen tissues were immediately fixed in 10% buffered formalin for 24 h and embedded in paraffin. Tissue sections (4 μm thicknesses) were treated with 3% H2O2 diluted in Tris-buffered saline (TBS) (pH 7.4) for 30 min. For antigen retrieval, tissue sections were immersed in citrate buffer (pH 6.0) for 20 min at 95°C. For detection and quantification of DENV-infected cells in spleen, an anti-DENV NS3 MAb (Gene Tex) or an isotype control was used with a dilution of 1:50 at 4°C overnight in a humidified chamber. After incubation, tissue sections were washed with TBS and treated with a labeled streptavidin-biotin kit EnVision + Dual Link System-HRP (Dako). Sections were then rinsed in PBS with 3,3′-diaminobenzidine tetrahydrochloride (K3468, Dako) for 5 min and stained with Mayer’s hematoxylin. For quantification of NS3+ cells, cells counts were performed in 20 alternate microscopic high-power fields (x 400) for each sample (4–5 mice per group). The number of positive cells in each field in the red pulp of spleen was counted and the mean calculated. Areas from the white pulp were excluded from analysis.

### Statistical analysis

All calculations were done using GraphPad Prism v5.0 (GraphPad Software Inc.).

## Supporting Information

S1 Fig DENV2 egress is dependent on proteasome function. (A) As a control, RNase treatment did not degrade mature packaged DENV2 from cell culture supernatant. Mean ± SD. N = 4. (B) Amount of GAPDH in the cells was significantly reduced after RNase treatment, demonstrating the effectiveness of RNase treatment in the removal of intracellular RNA. Mean ± SD. N = 4. Student’s t test, ***p<0.001.
(TIF)

S2 Fig MTS assays. (A) No cytotoxicity was observed in THP-1 cells after thapsigargin treatment using a cell viability assay. Mean ± SD. N = 4. (B) No cytotoxicity was observed in primary monocytes after bortezomib treatment using a cell viability assay. Mean ± SD. N = 4. (C) No cytotoxicity was observed in primary monocytes after epoxomicin treatment using a cell viability assay. Mean ± SD. N = 4.
(TIF)

S1 File Supplementary Materials and Methods and tables. (DOCX)
