# Supplementary figures and images for: Proteasome Inhibition Suppresses Dengue Virus Egress in Antibody Dependent Infection
Source: PLoS Negl Trop Dis. 2015 Nov 13;9(11):e0004058. doi: 10.1371/journal.pntd.0004058 (PMC4643959; doi:10.1371/journal.pntd.0004058)

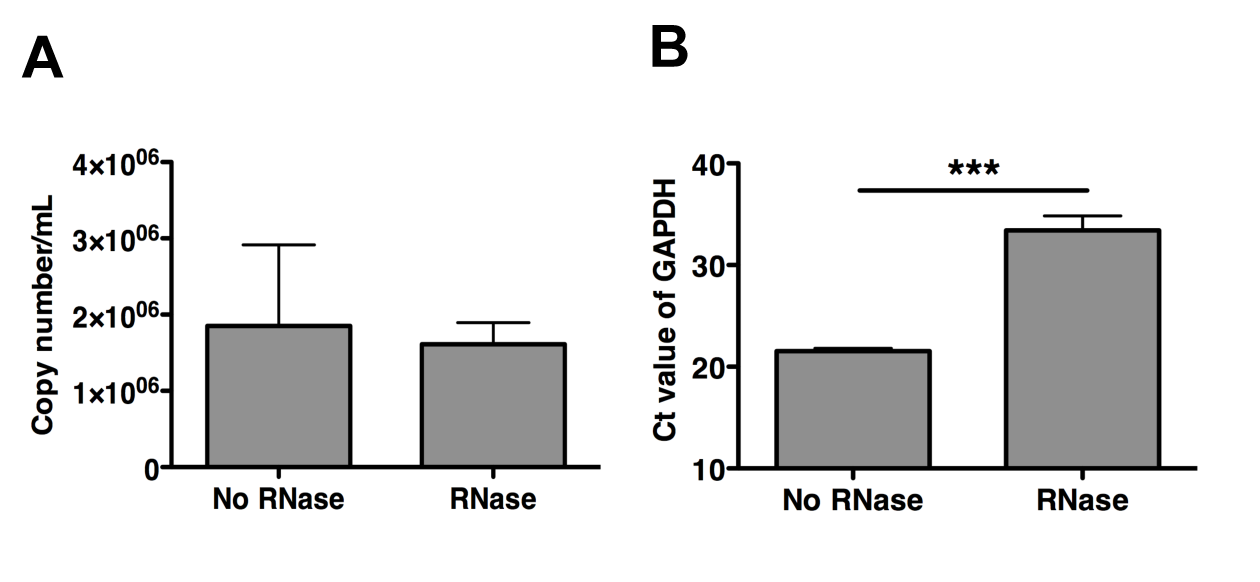

Supplement: S1 Fig — (A) As a control, RNase treatment did not degrade mature packaged DENV2 from cell culture supernatant. Mean ± SD. N = 4. (B) Amount of GAPDH in the cells was significantly reduced after RNase treatment, demonstrating the effectiveness of RNase treatment in the removal of intracellular RNA. Mean ± SD. N = 4. Student’s t test, ***p<0.001. (TIF) [file pntd.0004058.s001.tif]

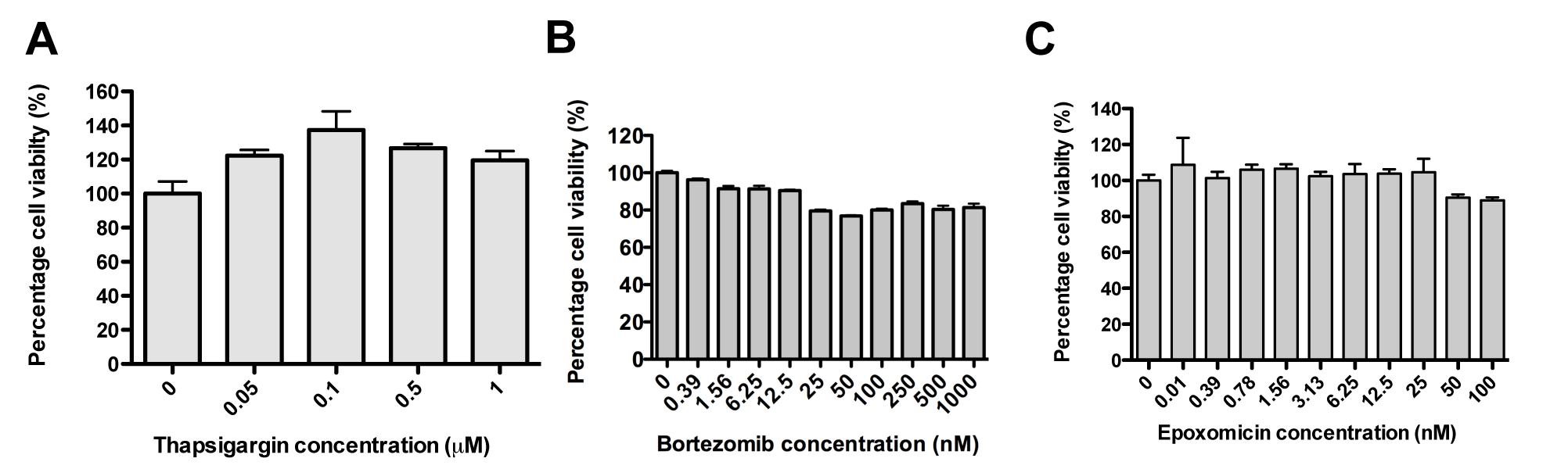

Supplement: S2 Fig — (A) No cytotoxicity was observed in THP-1 cells after thapsigargin treatment using a cell viability assay. Mean ± SD. N = 4. (B) No cytotoxicity was observed in primary monocytes after bortezomib treatment using a cell viability assay. Mean ± SD. N = 4. (C) No cytotoxicity was observed in primary monocytes after epoxomicin treatment using a cell viability assay. Mean ± SD. N = 4. (TIF) [file pntd.0004058.s002.tif]
