# Supplementary material for: Proteasome Inhibition Suppresses Dengue Virus Egress in Antibody Dependent Infection
Source: PLoS Negl Trop Dis. 2015 Nov 13;9(11):e0004058. doi: 10.1371/journal.pntd.0004058 (PMC4643959; doi:10.1371/journal.pntd.0004058)
Supplement: S1 File — (DOCX) [file pntd.0004058.s003.docx]

**Supplementary Materials**

**Primary monocytes isolation**

Venous blood from the principal investigator was collected in BD sodium heparin vacutainers (Biomed Diagnostics). The blood was then diluted with 2 volumes of 0.5% BSA (Sigma Aldrich) in phosphate buffered solution (PBS, 1st Base) (0.5% PBS/BSA) and carefully layered onto Ficoll-hypaque (GE Healthcare). The blood was then centrifuged at 750 × g, without brakes. The interphase cells containing the peripheral blood mononuclear cells (PBMCs) were aspirated and transferred to a clean tube. The PBMCs were washed three times with 0.5% PBS/BSA and resuspended in growth medium (RPMI-1640 supplemented with 10% FBS, 100 U/mL penicillin, and 100 μg/mL streptomycin). The cells were then seeded into T75 tissue culture flasks (NUNC) at 3 ×10^7^/flask and incubated at 37°C, 5% CO2 for 2.5 h, to allow plastic adherence of the monocytes to the flask surface. The adhered monocytes were washed five times with PBS to remove the non-adherent lymphocytes and replenished with fresh growth medium. These monocytes were allowed to recover overnight at 37°C, 5% CO_2_, before use in experiments.

**Virus stock**

DENV1-4 strains used in this study were propagated in the Vero or C6/36 cell lines and harvested when 75% or more of the cells showed cytopathic effect. Infectious titer was determined by plaque assay. To obtain high virus titer, the virus was purified through 30% sucrose cushion as previously described [[1](#_ENREF_1)]. Virus pellets were resuspended in 5 mM Hepes, 150 mM NaCl, and 0.1 mM EDTA (HNE) buffer and stored at −80 °C until use. Infectious titer was determined by plaque assay.

**Plaque assay**

Serial dilutions (10-fold) of virus were added to BHK-21 cells in 24-well plates and incubated for 1 hour at 37 °C. Media was aspirated and replaced with 0.8% methyl-cellulose in maintenance medium (RPMI-1640, 2% FCS, 25 mM Hepes, penicillin, and streptomycin). After 5 days at 37 °C, cells were fixed with 20% formaldehyde at room temperature for 20 min and washed with water, and 1 mL of 1% crystal violet was added for 20 min. The plates were washed and dried, and PFU/mL were calculated.

**qRT-PCR**

Total RNA was isolated using RNeasy® Mini kit (Qiagen) and reverse transcription was performed using the SuperScript™ III First-Strand Synthesis System. qRT-PCR was carried out using SYBR Green PCR Master Mix and the LightCycler® 480 System (Roche) using the primers listed in S1 Table. Expression values were normalized against GAPDH and technical duplicates were run for each sample.

**MTS cell viability assay**

Cells were treated with PBS, DMSO, β-lactone, genistein, thapsigargin, bortezomib or epoxomicin as per experimental conditions. 20 µL of CellTiter 96® AQueous One Solution Reagent (Promega) was added into each well of the 96-well assay plate containing the samples in 100 μL of culture medium. The plate is then incubated for 1-4 hours at 37°C and absorbance read at 490 nm.

**DiD labeling of DENV2**

DiD labeling of DENV2 was performed as previously described [[1](#_ENREF_1)]. Briefly, ~2.8 × 10^8^ PFU DENV2 was mixed with 800 nmol of DiD (1, 1′-dioctadecyl-3, 3, 3′, 3′-tetramethylindodicarbocyanine, 4-chlorobenzenesulfonate salt, Invitrogen) in DMSO (final DMSO concentration <2.5%). After 30 min, free DiD was removed by gel filtration on a Sephadex G-25 column (Amersham Biosciences) equilibrated in HNE buffer. DiD-labeled DENV2 was stored at 4°C and used within 24 hours.

**Alexa Fluor labeling of DENV2**

As previously described [[5](#_ENREF_5)], ~9 × 10^8^ PFU of DENV2 was labeled with 100 μM of Alexa Fluor 647 succinimidyl ester (Molecular Probes and Invitrogen) for 1 hour at room temperature. The labeling reaction was then stopped by adding 1.5 M hydroxylamine, pH 8.5, and incubated at room temperature for 1 hour. The excess dye was then removed by gel filtration on a Sephadex G-25 column. AF647- DENV2 was stored in 100 μL aliquots at −80 °C, retitrated by plaque assay, and tested for fluorescence using immunofluorescence assay on Vero cells before use in experiments.

**Western blots**

THP-1 cells are pretreated with stated concentrations of β-lactone, thapsigargin or DMSO and harvested at 4 or 24 hours. For infection, h3H5-DENV2 complexes were added after pretreatment with drugs. Cells were washed once in PBS and resuspended in lysis buffer containing 1% Nonidet P-40, 150 mM NaCl, and 50 mM Tris, pH 8.0. The cell lysates were clarified by centrifugation, separated by SDS/PAGE gel and probed for NS3 (3F8 antibody), E (HB112, ATCC), EXOC1 (Abcam), EXOC7 (Abcam), TC10 (Abcam), phospho-eIF2α (Cell Signaling), total eIF2α (Cell Signaling), BiP (Abcam), GAPDH (Abcam) and β-actin (Cell Signaling), followed by addition of anti-human or anti-mouse (Dako) or anti-rabbit (Abcam) IgG–horseradish peroxidase. Bands were visualized using ECL (Amersham) for chemiluminescence development.

**Immunofluorescence**

THP-1 cells pretreated with DMSO or 20 µM β-lactone and infected with h3H5-DENV2 complexes (moi 10) for 24 hours were harvested and fixed in 3% paraformaldehyde. The cells were cytospinned onto slides, permeabilized with 0.1% saponin in PBS containing 5% BSA for 30 min and stained for E (HB46, ATCC), prM (HB114, ATCC), Golgi (GM130, Abcam) and DAPI. The samples were then viewed with Zeiss LSM 710 confocal microscope.

**Transmission Electron Microscopy**

THP-1 cells pretreated with DMSO or 20 µM β-lactone and infected with h3H5-DENV2 complexes (moi 10) for 24 hours were harvested and fixed with 2.5% gluteradehyde + 2.5% paraformaldehyde in PBS, pH 7.4 for 2 hours at room temperature. Cells were then post-fixed with 1% osmium tetraoxide, dehydrated in a series of ethanol and embedded in Spurr’s resin. Samples were cut into ultrathin sections, stained with uranyl acetate followed by lead citrate and viewed using electron microscope JEM 2010F.

**Measurement of hematocrit level and platelet count in whole blood**

The mice were euthanized in a CO2 chamber and whole blood was collected using a 27-gauge needle fitted to a 1 ml syringe via cardiac puncture. During all time point, hematocrit level and platelet count in whole blood were measured. Hematocrit levels were quantified in the Veterinary Diagnostic Laboratory at MD2 (NUS). Platelets were quantified by flow cytometer analysis. Briefly, to count platelets 10 μL of whole blood from mice was mixed with 600 μL of FACS buffer solution (PBS containing 0.2% of bovine serum albumin (BSA) and 0.05% of sodium azide). Antibody specific for mouse platelets (anti-mouse CD41 clone MWReg30 from Biolegend Int) was added and incubated for 30 min. A total of 25 μL of CountBright absolute counting beads (Invitrogen) was added to each sample and analyzed until 1000 beads recorded events per sample. The number of platelets was calculated as follows: count of platelets/μL of whole blood = (mouse platelet events/ beads events) X (bead count/10 μL). Flow analysis was performed using a LSRII flow cytometer (Becton Dickinson).

**Viral load quantification in mouse spleen**

For viral load quantification, 30 mg of spleen was collected and stored in RNAlater RNA stabilization reagent (Qiagen) at -20^o^C. Spleens were homogeneized using stainless steel beads (5 mm) in Qiagen TissueLyser LT. The homogenate was collected, and viral RNA extraction for qRT-PCR was performed**.** Viral RNA was extracted from spleen using the RNeasy Mini Kit (Qiagen). Reverse transcription and amplification was done using the one-step quantitative reverse-transcription-PCR (qRT-PCR)-based QuantiFast probe RT-PCR Kit (Qiagen) with forward and reverse primers of the E gene in S1 Table, along with the 6- carboxyflurescein (FAM)-conjugated probe sequence CGATGGAATGCTCTC. The synthetic DNA containing the sequences containing both primers and the probe (5’-ACACCACAGAGTTCCATCACAGAAGCAGAACATACAGGCTATGGCACTGTCACGTGGAATGCTCTCCGAGAACGGGCCTCGACTTCAATGAGATG-3’) was serially diluted to achieve the standard curve. qRT-PCR was performed using the CFX96 real-time system (Bio-Rad). The sensitivity of the qRT-PCR was around 100 copies of viral RNA.

| Gene | Forward Primer (5’ to 3’) | Reverse Primer (5’ to 3’) |
| --- | --- | --- |
| DEN2 3’UTR | TTGAGTAAACYRTGCTGCCTGTAGCTC | GAGACAGCAGGATCTCTGGTCTYTC |
| DEN2 Envelope | ACACCACAGAGTTCCATCACAGA | CATCTCATTGAAGTCCNAGGCC |
|  |  |  |
| Human GAPDH | GAGTCAACGGATTTGGTCGT | TTGATTTTGGAGGGATCTCG |
| Human EXOC1 | CCTGTGCAGGTTAAGGTGGT | TTGGCATCTACCACAGCAAG |
| Human EXOC7 | CTGCTATCCCCAACAAGAGG | GTGGATGTAGGCATCGGTCT |
| Human TC10 | TTCGACCACTACGCAGTCAG | AAAGGCCTCAGACGGTCATA |

**S1 Table. Primers for qPCR.**
